# Talking Cancer—Cancer Talking: A Linguistic and Thematic Analysis of Patient Narratives

**DOI:** 10.1177/23743735241309472

**Published:** 2024-12-23

**Authors:** Ad A Kaptein, Pim B van der Meer, Fleur L Fisher, Hanneke W M van Laarhoven, James W Pennebaker, Ad J J M Vingerhoets

**Affiliations:** 1 Department of Medical Psychology, 4501 Leiden University Medical Centre, Leiden, the Netherlands; 2 Department of Psychiatry, University Medical Centre Utrecht, Leiden, the Netherlands; 3 Department of Neurosurgery, Leiden University Medical Centre, Leiden, the Netherlands; 4 Department of Medical Oncology, 522567 Amsterdam University Medical Centres, Location University of Amsterdam, Amsterdam, the Netherlands; 5 Cancer Center Amsterdam, Cancer Treatment and Quality of Life, Amsterdam, the Netherlands; 6 Department of Psychology, 12330 The University of Texas at Austin, Austin, TX, USA; 7 Department of Medical and Clinical Psychology, 7899 Tilburg University, Tilburg, the Netherlands

**Keywords:** patient narratives, LIWC (Linguistic Inquiry and Word Count), thematic analysis, cancer, illness perceptions, health humanities

## Abstract

To explore “the lived experience” of patients with cancer through narratives, in-depth interviews with 20 patients were conducted in the patients’ homes—“at the kitchen table.” Interviews were audio-recorded, transcribed, and analyzed following the Linguistic Inquiry and Word Count (LIWC) methodology. Thematic Analysis was used to explore themes in the narratives. Scores on relevant LIWC dimensions of the 20 patients were compared with norm data for respondents without cancer. Patients with cancer scored higher on “anger” and “sadness” (psychologic processes dimension); lower on “insight,” “causes,” and “tentatives” (cognitive processes dimension); and lower on “religion.” Major themes identified from the Thematic Analysis were resilience, fatigue, social relationships, turning inward psychologically, shared decision-making, and psychological support. Narratives of patients with cancer are a source of rich data on how persons with cancer make sense of their illness, its medical management, and its psychological and social consequences. Qualitative methods of data analysis (LIWC; Thematic Analysis) are a highly valuable element in the methodology of exploring patient experience.

## Introduction

How patients experience illness is a crucial topic of study in research and clinical care (eg, Health Humanities).^1,2^ In the field of oncology, scientific societies emphasize the importance of identifying and addressing patient experiences.^3,4^ Furthermore, scientific journals—medical and biopsychosocial alike—advocate the need to focus attention on the patient experience.^5–7^ In this journal, Meadows 2021^8^ in his paper “Patient-reported outcome measures—A case for more narrative evidence,” emphasizes the value of listening to the patient's story.

Traditionally, the psychological response to cancer is assessed using questionnaires with known psychometric characteristics (eg, EORTC-QLQ-C30, HADS, and SF-36) and various cancer diagnosis-specific questionnaires.^9–12^ Advantages of using questionnaires include the possibility to calculate a score and make comparisons between various patient samples, the patient-friendly format and length, and contributing to the body of knowledge on patient experience in the oncology field^13,14^ (in this Journal). However, measures of quality of life (QOL) and the related concept of patient-reported outcome have a significant limitation: the categories are defined by the researcher. This implies that, as Meadows rightly emphasizes: “… the patient's illness narrative is lost along the way … being restricted by the frame offered by the instrument and researcher”.^8^ Overwhelming evidence shows that a large gap exists between how patients define QOL and how health care providers define QOL.^15^ Health care providers, by definition, are the lay persons when it comes to patients’ QOL.

Limitations of quantitative questionnaires can be mitigated by using methods of qualitative research. Qualitative approaches in studying patients’ experience allow patients to tell their illness narratives; it allows them “to let their stories breathe”,^2^ rather than being forced to cross boxes in questionnaires designed by researchers who tend to be quite removed from the lived experience of patients. “The words that people use in everyday life tell us about their psychological status: their beliefs, emotions, thinking habits, lived experiences, social relationships, and personalities”^16, p. 2^.

Various methods of studying the patient's lived experience to cancer and its medical management can be found in the field of health humanities; bibliography, expressive writing, drawing, graphic novels, photo voice, painting, and performing music are the most frequently used and studied.^11,17–21^ Health humanities attempt to circumvent the phenomenon of social desirability; health humanities may also discourage patients adopting a biomedical narrative that tends to obscure the lived reality of having cancer and having to cope with the illness and its medical management.

Thematic analysis is a qualitative approach to uncovering and categorizing themes in spoken or written utterances of respondents.^22^ Another qualitative approach in examining the lived experience is to use the Linguistic Inquiry and Word Count (LIWC) methodology to examine words and written text by patients with cancer. One of the first studies in this area was conducted by Smith et al in the United States, where patients with breast cancer kept diaries about their illness and its biopsychosocial concomitants.^23^ A simple literature search in PubMed using the search terms “LIWC AND cancer” [up to 16 May 2024] identified 17 empirical studies where the LIWC methodology was used to analyze the impact of cancer on various aspects of QOL in patients. An overview of these studies is presented in Table 1.

**Table 1.** Empirical Studies With Linguistic Inquiry and Word Count (LIWC) Methodology in Patients With Cancer, Identified in the PubMed Search “LIWC AND Cancer” (16 May 2024).

First author, year, country	Number of patients, diagnostic category	Dependent variable(s)	Results
Arch, 2022, USA^17^	n = 88, breast cancer	Adherence with endocrine therapy	“Emotion words” (low levels of anger, anxiety, sadness) associated with adherence
Badr, 2016, USA^18^	n = 123, head and neck cancer	Quality of marital adjustment	Emotion words and personal pronouns associated with quality of marital adjustment
Bantum, 2009, USA^19^	n = 63, breast cancer	Emotional expression in written text in intervention study format	LIWC identifies emotional expression more than human raters
Borelli, 2022, Italy^20^	n = 133, patients in early palliative care setting	Gratitude for care	Gratitude words associated with communication and spirituality are related to quality of life
Crook, 2016, USA^21^	Online YA (young adolescent) cancer support community	Posts with or without a reply	More replies are elicited by first-person singular words and expressed emotions; posts without replies include more words per sentence
Kaal, 2018, The Netherlands^22^	n = 30, AYA's (adolescents and young adults) with cancer in online support group	Usefulness of the support group	Patients’ LIWC data report emotional support, information exchange and coping as helpful
Laccetti, 2007, USA^23^	n = 68, women with metastatic breast cancer	Quality of life in expressive writing study	Positive-affect word use associated with disclosure and quality of life
Lewallen, 2014, USA^24^	n = 116, members of online cancer support group	Replies to posts	Posts including self-disclosure, medical experiences and social relations elicit responses
Liaw, 2022, Malaysia^25^	n = 31, patients with cancer	Cognitive and emotional responses to cancer	More use of cognitive than affective words; more use of positive than negative emotion words
Liess, 2008, USA^26^	n = 36, women with breast cancer	Compare written text analysis with video coding of patients in support groups	Discordance between LIWC and human coders of emotional expression
Martino, 2015, Italy^27^	n = 20, women with breast cancer	Effects of expressive writing	Increases in positive emotion words, greater use of cognitive processing words
McDonnell, 2020, USA^28^	n = 63, women with breast cancer	Internet-based support group	Emotional expression identified similarly by human coders and LIWC
Owen, 2006, USA^29^	n = 71, community cancer support group	Expressive writing about cancer	Emotional suppression associated with distress
Smith, 2005, USA^16^	n = 43, women with breast cancer	Effects of expressive journal writing on mood	Writing about negative emotions is associated with increased anxiety and depression
Verberne, 2019, The Netherlands^30^	n = 2071, users of forum for persons with cancer	Empowerment processes in communication	Words expressing assent and emotional processes predict emotional support
Williamson, 2017, USA^31^	n = 67, stem cell transplant recipients	Expressive helping and expressive writing essays	Expressive helping reduces distress through expression of positive emotions
Wu, 2023, China^32^	n = 112, women with breast cancer	Expressive writing (short, 4 weeks) vs long (12 weeks)	Level of emotional expression determines quality of life

Table 1 summarizes the 17 empirical studies where LIWC methodology was applied in patients with cancer, and the associations of various LIWC dimensions with various aspects of QOL in persons with cancer.^24–39^

The majority of studies are from the United States, with breast cancer as the diagnostic category studied most often. Sample sizes vary from 20 to 2071.^27,30^ The results are in line with research on the associations between LIWC characteristics and illness behavior in persons with cancer: expressive writing in various forms and formats is associated with stronger use of emotion words, and with increased QOL scores.

## Methods

This study was conducted in the context of the “At the Kitchen Table” column in a journal for cancer professionals, *Medische Oncologie* (“Medical Oncology”) in the Netherlands (circulation 4000, 10 issues per year). Oncologists on the journal's editorial board asked patients in their outpatient clinics if they would like to participate in interviews about living with cancer. After informed consent was obtained, interviews were conducted in patients’ homes with the interviewer listening to their stories in an encouraging and supportive way. An open interview schedule was used, inspired by the paper by Mathieson and Stam,^40^ avoiding any attempt by patients to medicalize their situation (Table 2). In addition to the spoken text, patients provided “an object” (eg, a painting, a music piece, a sculpture, a poem, or a picture of the patient's children) to be published with the interview in the journal, that in their view represented their illness and its medical management.

**Table 2.** Schedule of Questions Asked During the Interviews (Adapted from Mathieson and Stam^40^).

Order	Question
1	Please think back to the 6 months preceding your diagnosis. How would you describe those months?
2	Describe to me what you recall thinking at the time of the initial diagnosis.
3	I would like you to summarize the course of your disease for me thus far. For example, can you tell me about your treatment? How would you describe your “normal week”? Describe to me what will happen in terms of your next course of treatment.
4	Do you think you have received/are receiving adequate information from health care professionals?
5	What things are different about your life now than before you had cancer?
6	How have people responded to your cancer? What have they said, or done, which was helpful, or not helpful? (a) spouse, (b) family, (c) close friends, (d) other friends, (e) coworkers, (f) health care professionals, and/or (g) anyone else? Did any of these things change the way you thought about yourself or about having cancer?
7	What is different about yourself since your diagnosis? In other words, is the way you see yourself now different from the way you saw yourself in the past?
8	What is different about your body since your diagnosis?
9	Since your diagnosis, has your relationship changed with your (a) partner, (b) family/children, and/or (c) friends?
10	What do you think caused your cancer?
11	What does the term the “future” mean to you right now? What are your feelings about your life expectancy?
12	Do you feel your cancer is/can be cured?
13	Is there anything that I have failed to ask you in this interview which is important for me to know?
14	Looking over your whole cancer experience, what is the most significant change in your life that has taken place as a result of the diagnosis?

All interviews were audiotaped, transcribed, and formatted into a Word document. The formatted transcripts were then analyzed using LIWC methodology and Thematic Analysis. Linguistic Inquiry and Word Count methodology is a nonobtrusive method of studying narratives of patients and of anyone who produces written or spoken text. With LIWC, various core characteristics in spoken or written narratives can be studied: emotion words, cognition words, utterances related to biological processes, drives, and some grammar characteristics.^16^ Also, words produced are analyzed by comparing linguistic characteristics of those words with dictionaries, produced by the LIWC developers and researchers.^41^ An example of how examining the use of words in the context of medical treatment of cancer is from Fridman et al: they found that “physicians’ use of loss words was correlated with physicians’ recommendations for cancer treatment versus active surveillance, and that loss words in consultations were associated with patients’ choice of cancer treatment”^42, p. 38^. The LIWC analysis was performed using *LIWC2015*.^43^ Comparisons were made with data from a representative sample of Dutch respondents, which were reported in Dudău et al.^44^

Thematic analysis was used to uncover Mathieson and Stam themes in the transcribed narratives. In their recent critical review of thematic analysis, Braun and Clarke state how “TA methods typically involve procedures for coding and theme development, the output of which is a set of themes, the potential for researchers to focus on semantic/manifest (…) and/or latent (…) meaning, and some degree of theoretical flexibility in the application/use of the method”^22, p. 699^.

## Results

Twenty patients consented to being interviewed: 9 men and 11 women, age ranging from 28 to 75 years. Various diagnostic categories were represented in the sample. The average duration of the interview was 75 min.

Twelve themes were identified from the thematic analysis (see Table 3). These themes related to physical, psychological, and social aspects of life, and the care system.

**Table 3.** Themes Identified From the Thematic Analysis of the Patients’ Narratives.

Physical	Psychological	Social	Care System
Fatigue	Resilience	Relinquishing superficial relationships	Views on health care providers
Pain	Turning inward	Resigning from outward appearance (money, status, possessions)	Shared decision making
Sleep problems	Chemofog	-	-
-	Fear of recurrence	-	-
-	Death is not an overriding thought; practical arrangements about end of life become important	-	-

Regarding *physical* themes: fatigue, pain, and sleep problems were the dominant issues mentioned by the patients. “The chemotherapy made me extremely sick for a week and a half. Then I recovered somewhat, and had to have another series. I had hardly time to recuperate.”

The theme Fatigue appeared to be a prevalent and central phenomenon.

“I’m exhausted because of the chemo. I sleep 3-4 h every afternoon.”

Five *psychology*-related themes were identified.

*Resilience*: Nearly all patients expressed a strong wish and attempts to actively cope with their illness and its impact on their psychological, social, and medical existence. Resilience demonstrated as searching for information about the illness and its medical management, exploring options offered via patient organizations and the internet, adjusting health behaviors (eg, eating, drinking alcohol, physical activity, sleeping), and searching for existential enrichment.

“Two weeks after the diagnosis ‘incurable disease’, I thought ‘O.K., that's it, no more complaining, nagging and crying. These are the facts. Get back to your life’. How long the tunnel may be—may be 3, 5 or 10 years—you cannot live in the dark.”

“It sounds strange, I would have preferred to stay healthy, but I find this an extremely interesting period in my life. I want to know what's happening in me, in my body. Why do I have this pain? I prefer to have control.”

Turning inward psychologically refers to the tendency to withdraw from situations and events that were being perceived as superficial, and not contributing to major values in life such as unconditional love, honesty, meaning finding, beauty, rest, and silence.

“I observe that I am focusing on what's really important: turning inward.”

“Cancer leads to existential loneliness. The horrific loneliness in the suffering—it's almost impossible to tell others about that.”

“I have become more complacent. I’m enjoying my life more. I’m o.k. with how it is.”

Chemofog [chemotherapy-related cognitive impairment]: “…it takes an enormous amount of energy to keep up. It is hard to focus and think—it's like you can’t look at the world as it is, as if there is a curtain in front of it. I lost a part of myself on the way. I think my environment notices that. I’m no more my old self.”

Fear of recurrence was mentioned by virtually all patients: “with any unusual bodily experience, panic strikes: “Oh no, please do not let it be cancer again.”

Dying and death were discussed in most interviews, in a fairly straightforward manner: “I have prepared my life, my house, and my social existence for my death, which feels good.” All patients mentioned that they had made plans regarding the end of their life (eg, burial, music, distributing their cherished possessions).

A major finding pertained to a tendency to try and strengthen *social* bonds with close friends and relatives, with the associated loosening of ties with more distant persons in the social environment.

Superficial friendships were ended, the value of psychologically distant relatives and friends was reduced to virtually zero. Patients reported to be disappointed and sometimes angry by the self-centered responses from superficial acquaintances to the patient's cancer.

“Over the years the number of people with whom I am in touch has reduced significantly. Now I’ve some 15 real friends with whom I interact intensely. I cut out fringe around my social world.”

“The responses from my social environment are always the same … ‘Yes, unpleasant, I have an aunt who also has cancer, … you look good, you can do it. With my aunt, however, it was end of story soon …’.”

With regard to the *care system*, health care providers were perceived as highly positive. The supportive and listening skills of specialized cancer nurses were particularly appreciated as highly positive. Shared decision-making was a preferred way of patient–physician relationship although a few patients preferred the “doctor knows best” style.

“My oncologist and I decided about the next treatment together. So, I was asked again and again whether I was o.k. with the next step. My intuition was decisive. If I indicated ‘no further than this now,’ we halted the treatment temporarily. It was a continuous shared decision making.”

Although psychological help was not often sought, patients who did seek help from psychologists were positive over their work overall. Patient organizations were not perceived to be very helpful. Religion was hardly mentioned.

Means and standard deviations of selected LIWC dimensions of the patients in the current study are given in Table 4, together with means and standard deviations in the Dudău et al study. Compared to LIWC scores of healthy respondents, the patients with cancer scored higher on “anger,” “sadness,” and lower on “religion”; the patients reported lower scores on issues relating to time. Regarding cognitive processes, the “wounded storytellers” exhibited significantly lower scores on “insight,” “cause,” and “tentatives”—pointing at the heightened sense of cognitive confusion and increased search for meaning about the lived cancer experience.

**Table 4.** Scores (Means ± Standard Deviations) on Selected LIWC Dimensions in Current Study Compared With Scores in Dudău et al Study (Healthy Dutch Sample).^44^

LIWC dimension	Current study	Dudău et al study	Current study vs Dudău study
Affect			
Positive	2.47 ± 0.62	2.33 ± 1.08	
Negative	1.40 ± 0.44	1.12 ± 0.68	*
Anxiety	0.27 ± 0.19	0.22 ± 0.25	
Anger	0.39 ± 0.14	0.26 ± 0.29	***
Sadness	0.62 ± 0.29	0.29 ± 0.27	***
Cognitive processes			
Insight	2.38 ± 0.40	2.96 ± 0.88	***
Cause	1.02 ± 0.23	2.11 ± 0.75	***
Tentatives	3.09 ± 0.66	2.72 ± 0.84	*
Social			
Family	0.54 ± 0.35	0.53 ± 0.64	
Friends	0.16 ± 0.10	0.15 ± 0.17	
Time			
Past	4.91 ± 1.22	5.47 ± 1.77	
Present	11.29 ± 0.95	12.72 ± 2.10	***
Future	0.79 ± 0.21	2.29 ± 0.79	***
Personal concerns			
Religion	0.09 ± 0.16	0.17 ± 0.34	*
Death	0.16 ± 0.15	0.15 ± 0.25	

* *P *< .05.

** *P *< .01.

*** *P *< .001.

## Discussion

The results in this (18^th^) study using LIWC methodology to examine narratives of patients with cancer reveal that these patients with cancer show increased levels of negative affect, anger, sadness, an increased sense of cognitive confusion, a weaker focus on both future and present time orientations, and a weaker orientation on religion—in comparison to Dutch persons without cancer. Themes identified with thematic analysis corroborate these empirical findings, with an additional emphasis on resilience and psychological withdrawal from the part of the social world that is perceived as superficial.

A major contribution of our study relates to the narratives about the “turning inward” by the patients: turning away from money, status, possessions, and superficial social relationships, and directing their attention to the truly meaningful social relationships in their lives, together with attempts to give meaning to being ill and facing death. This finding aligns with the insights from the recently developed program “CALM–Managing Cancer And Living Meaningfully”^5^ that also emphasize the importance and relevance of spirituality when being a person with cancer.

Our findings are in line with previous studies. Our observation that resilience, turning inward, relinquishing superficial relationships, and focusing social life on persons close to the patient are core themes align with findings from a recent systematic review and meta-analysis of expressive writing, which highlights the relevance of studying the expression of cognitions and emotions by patients with cancer on health outcomes, that is, fatigue, passive mood, and physical aspects of QOL were positively affected, underscoring the issues identified in this study.^45^

### Limitations

There are 5 limitations of this current study. The first is the relatively small number of patients in our sample. This makes the current study a pilot study. Second, we are unaware of studies in other Dutch patient groups where LIWC2015 was used. This limits comparison of scores on LIWC dimensions in our sample with other samples. Third, the patients in the study may represent a somewhat unrepresentative sample: the patients were asked by their oncologist to participate in the study where the interview would focus on patient experience—not on blood values or MRI results—which may imply that the patients were able and willing to discuss personal matters, more than the “average” person with cancer. Fourth, themes in the Thematic Analysis method were identified by one assessor, although a second coauthor read the verbatims of the interviews of all patients.

Finally, an important limitation pertains to the LIWC data reported by Dudău. The words used in TED-talks that form the basis for those LIWC scores may differ from the possibly more emotional and personal words used by the patients in the study. In fact, the authors of the study themselves acknowledge that “The communication context could produce variation in the frequency of both content and function words, …” (p. 15), and also that “ … the translation process of the TED talks might have been another potential source of bias … ”^44, p. 15^. It is unclear if and to what degree these factors rather than the presence of the cancer diagnosis impacted LIWC scores. Future research on LIWC translations and norm data is warranted.

### Research Implications

Linguistic Inquiry and Word Count methodology can be an effective research tool to understand the context of wounded storytellers and their illness perceptions^46^: examining the way patients make sense of their illness is key to improving their QOL. Incorporating the study of words and language into research and clinical work may help identify novel approaches for research and clinical intervention.

### Clinical Implications

Modern research on supportive care for persons with cancer focuses on acknowledging the meaning given by patients to their cancer as operationalized in “illness narratives”,^2^ “explanatory models”,^46^ and “illness perceptions”.^47^ As such, the LIWC scores and themes identified in this study may be helpful in designing supportive care programs for persons with cancer. Another clinical implication relates to how the finding in our study on “turning inward” suggests the value of health care providers encouraging patients to make sense of the illness and attempt to weigh the quality versus quantity of their lives. Incorporating our findings and those from related relevant work into clinical guidelines for health care professionals could improve the care of patients with cancer.

## Conclusion

Listening to persons with cancer and analyzing their narratives with thematic analysis and LIWC methodology uncovers major themes in living with cancer, underlining the importance of supportive care for persons with cancer.
